# Supplementary figures and images for: Long non-coding RNA Malat1 fine-tunes bone homeostasis and repair by orchestrating cellular crosstalk and β-catenin-OPG/Jagged1 pathway
Source: eLife. 2024 Dec 23;13:RP98900. doi: 10.7554/eLife.98900 (PMC11666238; doi:10.7554/eLife.98900)

**Figure 3-Source Data 2. Original membranes corresponding to Figure 3 panel B**

Figure 3B

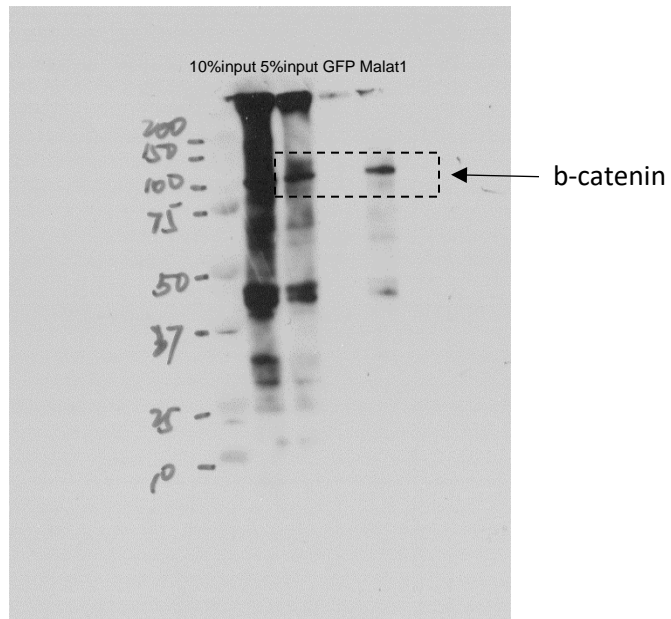

Figure 3D

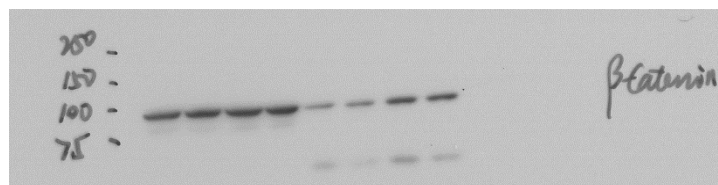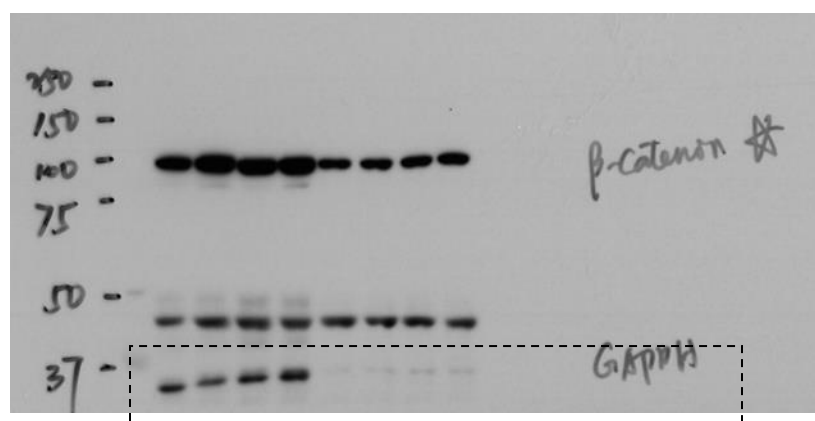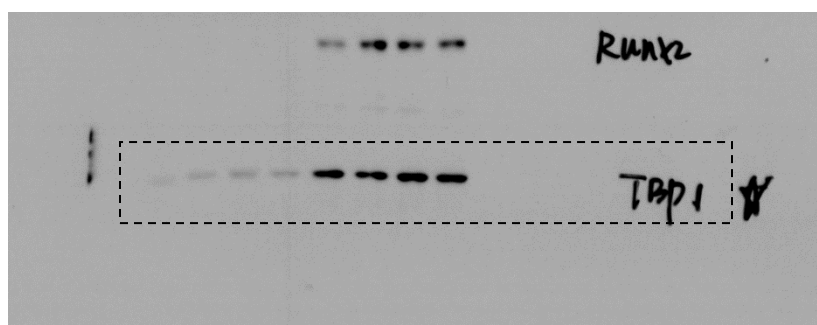

Supplement: Figure 3—source data 2. [file elife-98900-fig3-data2.pdf]

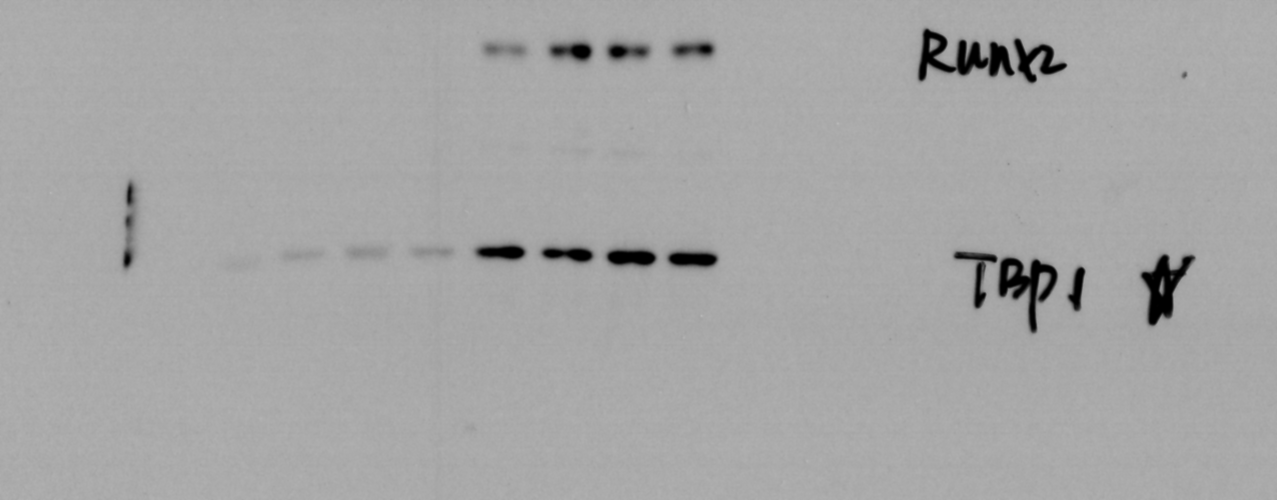

Supplement: Figure 3—source data 3. [file elife-98900-fig3-data3.zip › Figure 3D-TBP1.tif]

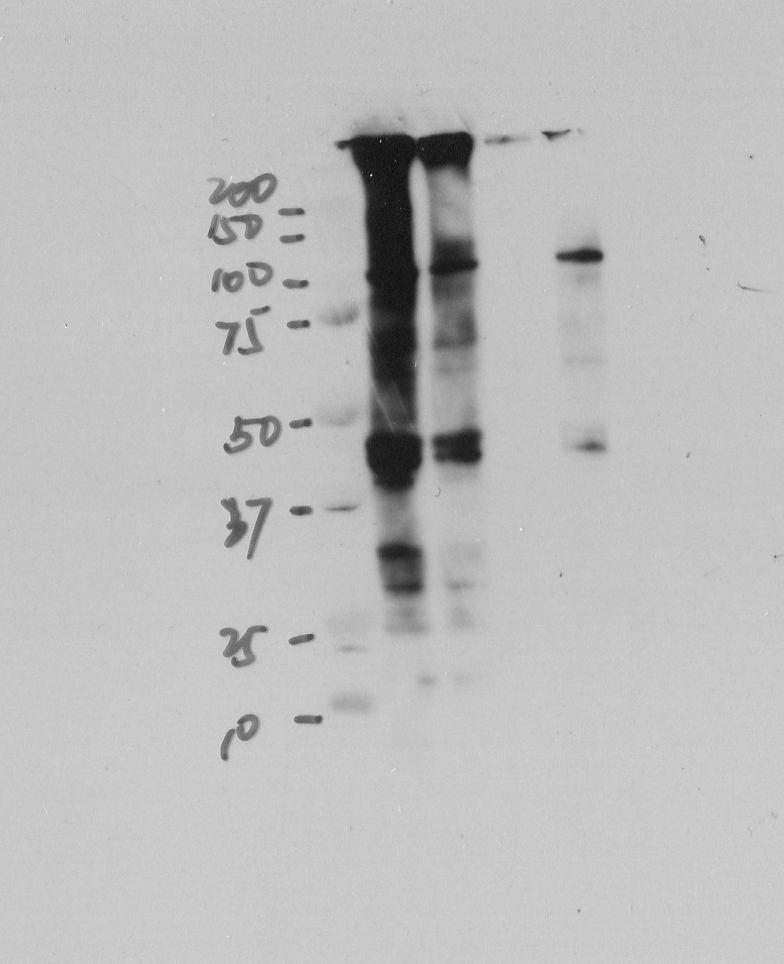

Supplement: Figure 3—source data 3. [file elife-98900-fig3-data3.zip › Figure 3B.tif]

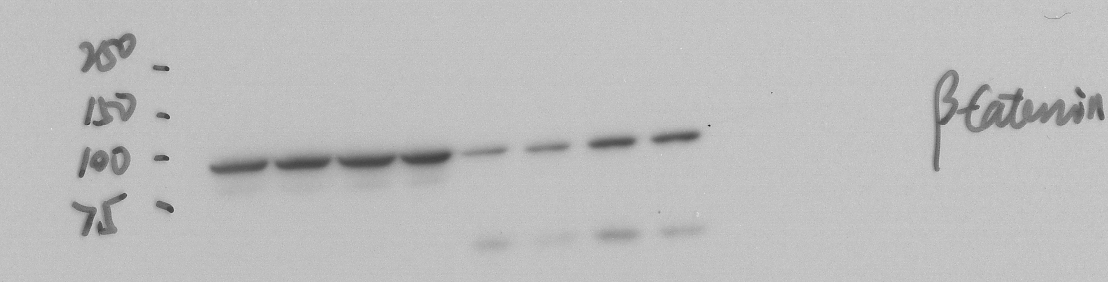

Supplement: Figure 3—source data 3. [file elife-98900-fig3-data3.zip › Figure 3D b-catenin.tif]

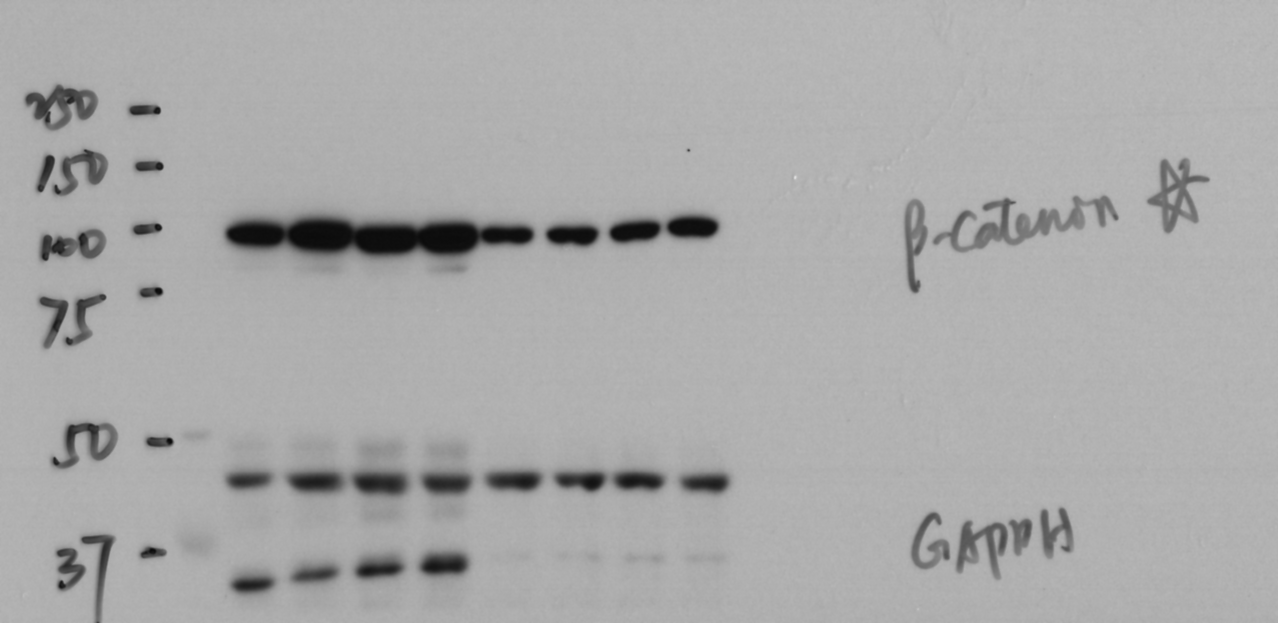

Supplement: Figure 3—source data 3. [file elife-98900-fig3-data3.zip › Figure 3D-GAPDH.tif]

Figure 4-Source Data 2. Original membranes corresponding to Figure 4 panel D

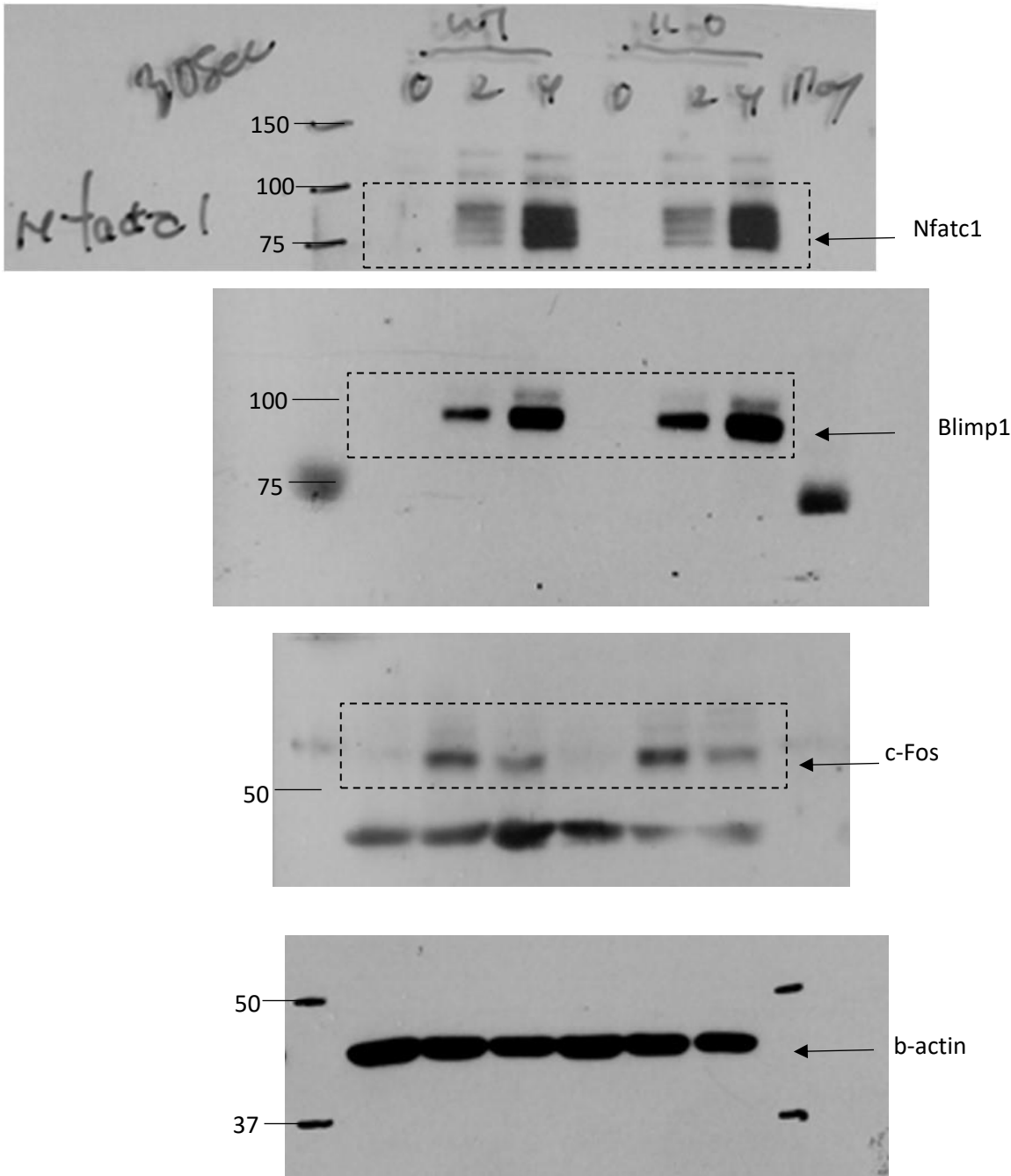

Supplement: Figure 4—source data 2. [file elife-98900-fig4-data2.pdf]

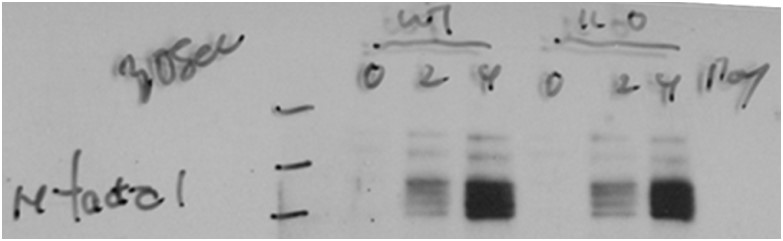

Supplement: Figure 4—source data 3. [file elife-98900-fig4-data3.zip › nfatc1.jpg]

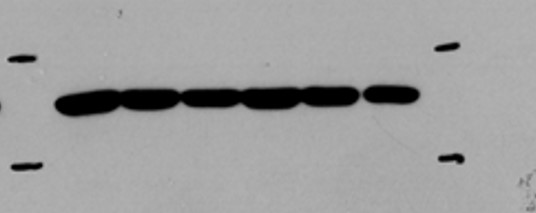

Supplement: Figure 4—source data 3. [file elife-98900-fig4-data3.zip › b-actin.jpg]

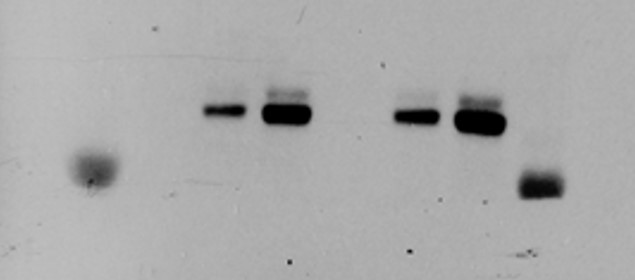

Supplement: Figure 4—source data 3. [file elife-98900-fig4-data3.zip › Blimp1.jpg]

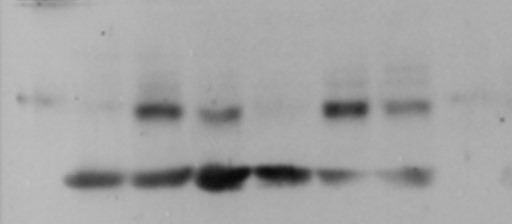

Supplement: Figure 4—source data 3. [file elife-98900-fig4-data3.zip › c-fos.jpg]

Figure 5-Source Data 2. Original membranes corresponding to Figure 5 panel H

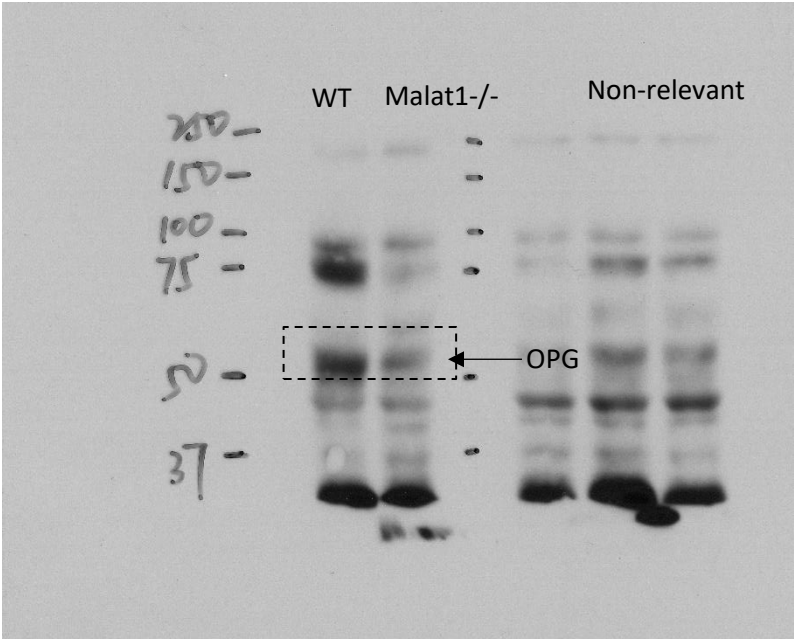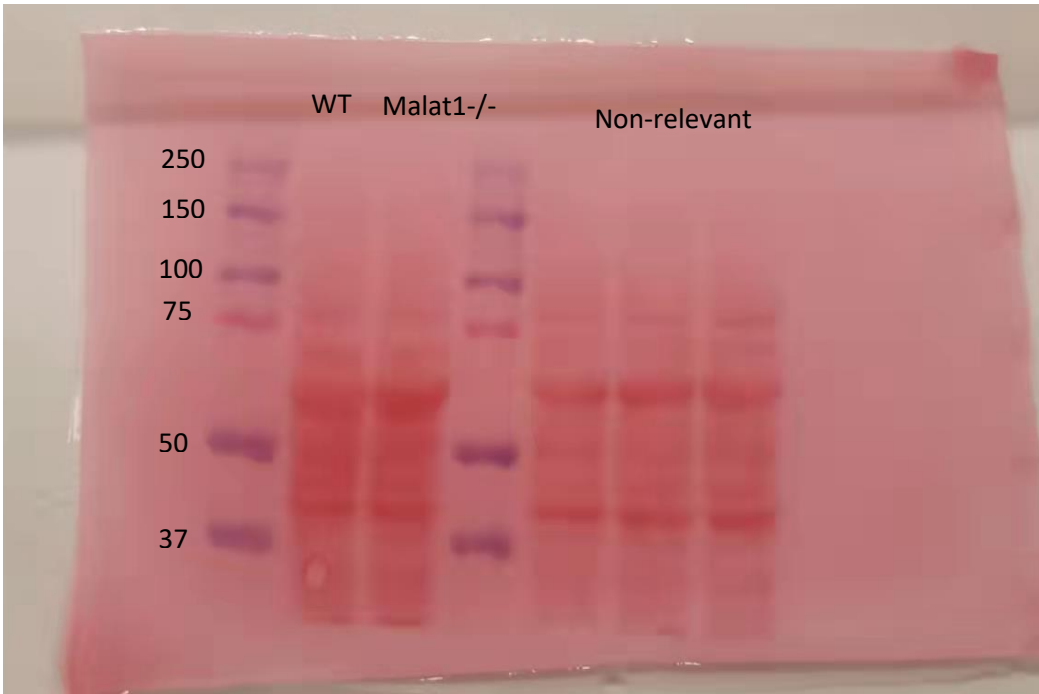

Supplement: Figure 5—source data 2. [file elife-98900-fig5-data2.pdf]

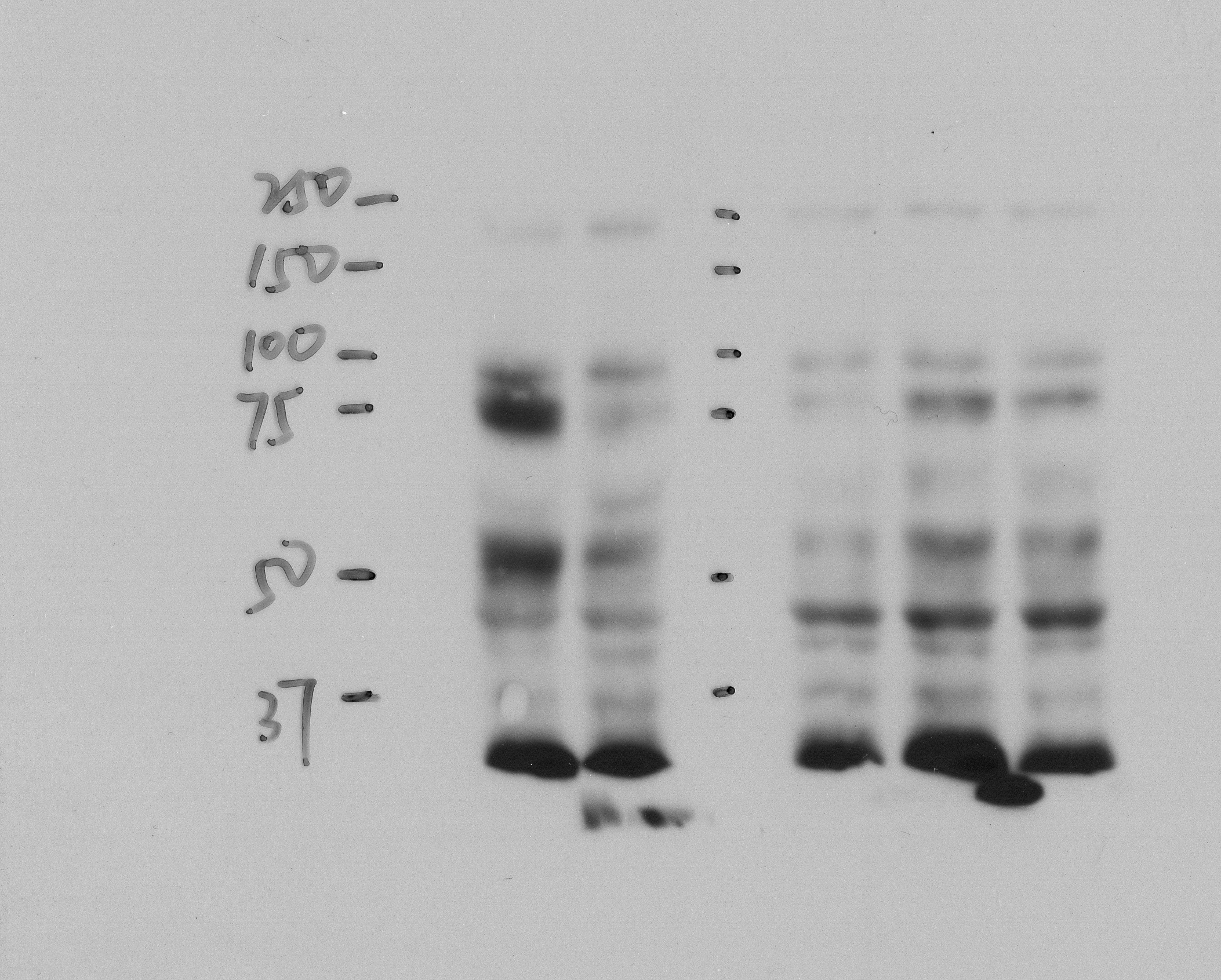

Supplement: Figure 5—source data 3. [file elife-98900-fig5-data3.zip › OPG.tif]

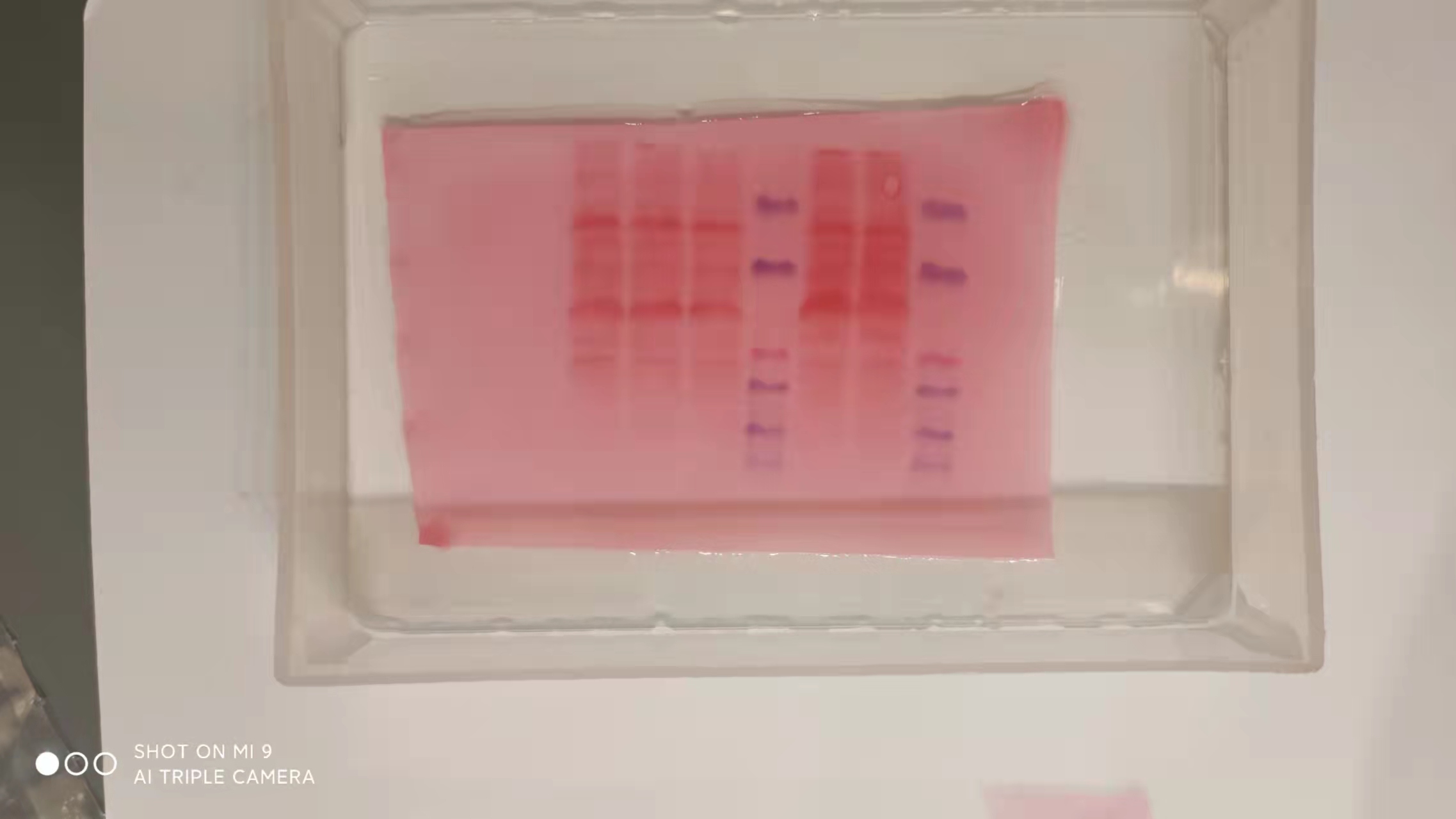

Supplement: Figure 5—source data 3. [file elife-98900-fig5-data3.zip › Ponceau Staining.jpg]

Figure 6-Source Data 2. Original membranes corresponding to Figure 6 panel G and I

Figure 6G

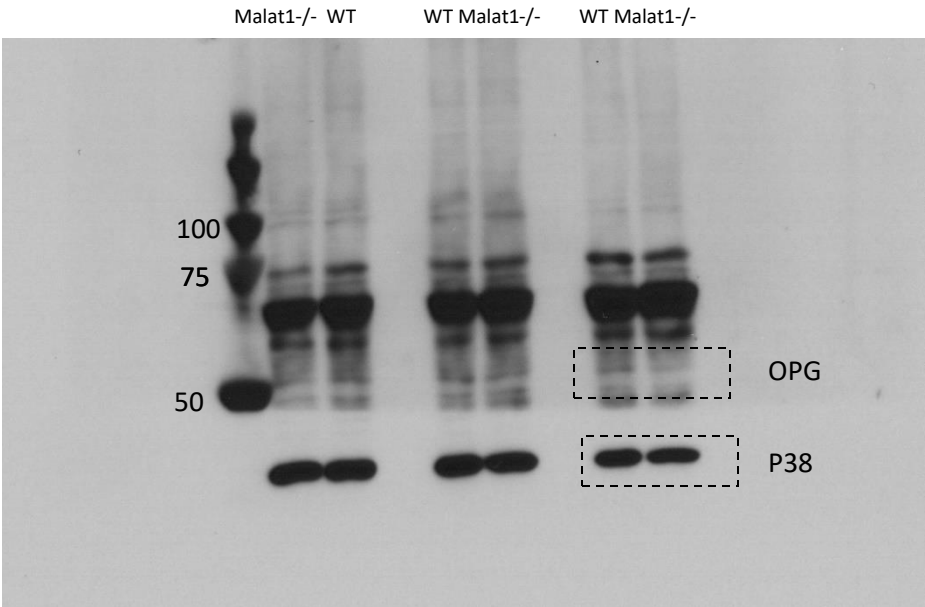

Figure 6I

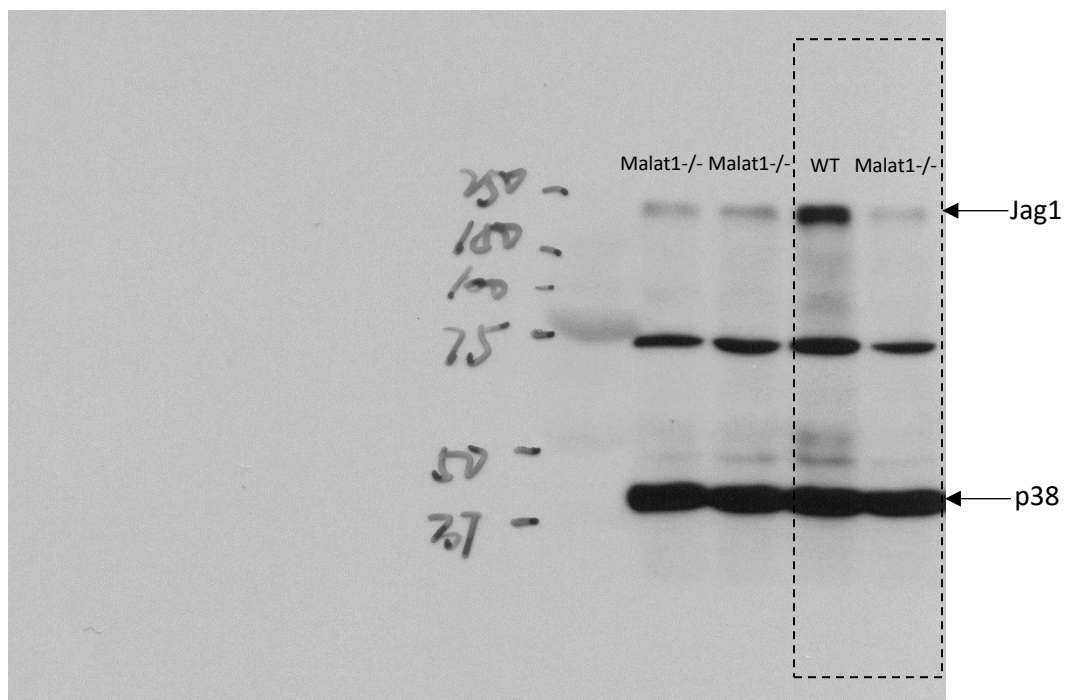

Supplement: Figure 6—source data 2. [file elife-98900-fig6-data2.pdf]

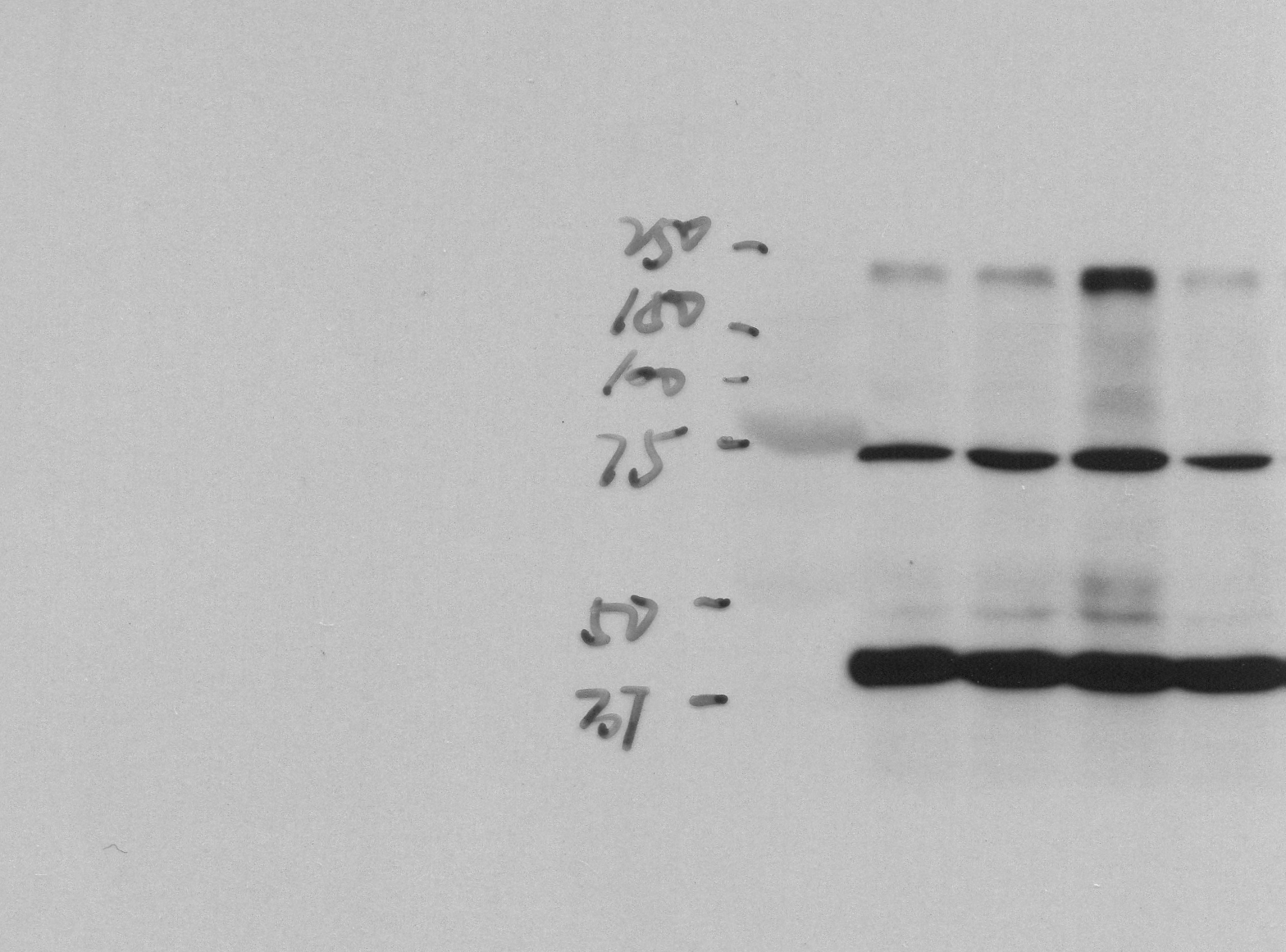

Supplement: Figure 6—source data 3. [file elife-98900-fig6-data3.zip › Figure 6I Jag1.tif]

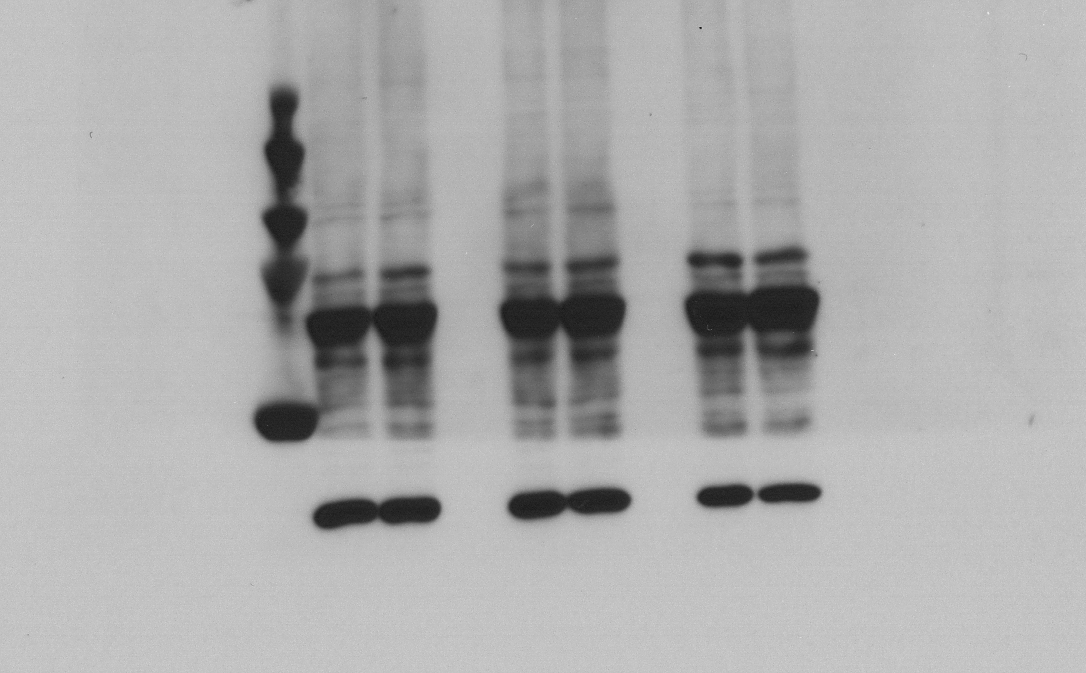

Supplement: Figure 6—source data 3. [file elife-98900-fig6-data3.zip › Figure 6G Opg.tif]
